# The Role of Hysterectomy in Modern Gynaecological Surgery

**DOI:** 10.1155/2022/9847163

**Published:** 2022-06-24

**Authors:** H. Krentel, R. L. De Wilde, G. Pados

**Affiliations:** ^1^Department of Gynecology, Obstetrics and Gynecological Oncology, Bethesda Hospital, Academic Teaching Hospital, Duisburg, Germany; ^2^Clinic of Gynecology, Obstetrics and Gynecological Oncology, University Hospital for Gynecology, Pius-Hospital Oldenburg, Medical Campus University of Oldenburg, Germany; ^3^1st Department of Obstetrics & Gynecology, “Papageorgiou” General Hospital, Aristotle University and Centre for Endoscopic Surgery “Diavalkaniko” Hospital, Thessaloniki, Greece

Hysterectomy plays a major role in gynecological surgery. The list of indications for hysterectomy is long and includes a variety of benign and malignant diseases. Over the last decades, new techniques allowed the implementation of new surgical approaches. Vaginal and abdominal hysterectomy has been complemented by laparoscopic procedures, such as vaginal hysterectomy assisted by laparoscopy, laparoscopic subtotal and total hysterectomy, and robotic hysterectomy. The implementation of new techniques was related to “new” complications, a series of studies comparing the alternative approaches for hysterectomy and an ongoing craftsmanship among gynecological surgeons about what is possible in laparoscopic surgery [1] [2] [3]. At the same time, a contrary movement dedicated to the preservation of the uterus emerged [4] [5] [6]. Hysterectomy was no longer the only solution in many uterine diseases, as techniques like radiofrequency ablation, uterine artery embolization, high-focused ultrasound, endometrial ablation, and minimally invasive tumor enucleation allowed uterus-sparing procedures in symptomatic patients. In this situation, we raised the question: what is the role of hysterectomy in modern gynecological surgery? G. K. Noé et al. focused on surgical techniques in pelvic floor disorders. The authors emphasized that hysterectomy requires its own indication and should not automatically be part of every pelvic floor intervention [7]. L. A. Torres-de la Roche et al. described a possible complication of uterine artery embolization and discussed the role of hysterectomy as a secondary intervention after treatment failure in uterus-sparing techniques [8]. While Q. Zhang et al. described the impact of B7-H4 expression in precancerous lesions of the uterine cervix and the decision-making process regarding follow-up and conization in patients with CIN2 [9], R. Wojdat et al. presented a retrospective analysis of their experience with vaginal assisted radical laparoscopic hysterectomy in patients with cervical cancer in the post-LACC trial era [10]. The role of hysterectomy in the management of obstetrical complications, especially in peripartum hemorrhage, has been reviewed by D. Tsolakidis et al. [11]. The broad spectrum of this special issue shows the relevance of hysterectomy in gynecological and obstetrical indications. Also, in modern gynecological surgery, hysterectomy plays a crucial role. But complete counseling of patients requires the consideration of all available hysterectomy approaches including vaginal, abdominal, laparoscopic, robotic, or combined techniques and the knowledge of uterus-sparing treatment alternatives. The aim of this special issue is to describe the significance of the uterus in a woman's life span and to highlight the actual trends in hysterectomy.
